# Perioperative risk stratification for periprosthetic joint infection after primary total knee arthroplasty: a case-matched cohort study incorporating serum albumin, glycemic status, and intraoperative hypothermia

**DOI:** 10.1186/s43019-026-00335-3

**Published:** 2026-07-10

**Authors:** Yung-Fong Tsai, Yu-Hong Ng, Shu-Yu Yeh, Yu-Hsun Sun, Huan-Tang Lin, Yu-Fang Liu, Shao-Chun Wu

**Affiliations:** 1 Department of Anesthesiology, Chang Gung Memorial Hospital, Linkou Branch, Taoyuan, Taiwan; 2 College of Medicine, Chang Gung University, Taoyuan, Taiwan; 3 Department of Anesthesiology, Kaohsiung Chang Gung Memorial Hospital, Kaohsiung, Taiwan; 4 Department of Anesthesiology, China Medical University Hospital, Taichung, Taiwan

**Keywords:** Hypothermia, Intraoperative temperature, Periprosthetic joint infection, Risk stratification, Total knee arthroplasty

## Abstract

**Background:**

Periprosthetic joint infection (PJI) remains a serious complication of primary total knee arthroplasty (TKA). This study investigated independent and combined associations of serum albumin, preoperative blood glucose, and intraoperative lowest body temperature (LBT) with PJI within 1 year after primary TKA.

**Materials and methods:**

This retrospective case-matched cohort study enrolled 312 patients (57 PJI cases, 255 controls) from 4319 screened undergoing primary TKA. Cases were matched 1:5 to controls by operating surgeon and year of surgery (frequency matching). Infection was defined by 2018 International Consensus Meeting criteria. Multivariable logistic regression (seven covariates, events-per-variable = 8.1) and receiver operating characteristic (ROC) analysis identified independent predictors and assessed discrimination. Two composite models were constructed using ROC-derived cutoffs: a two-factor preoperative model (serum albumin ≤ 4.1 g/dL + blood glucose ≥ 147 mg/dL) and a three-factor perioperative model additionally incorporating LBT ≤ 35.5 °C.

**Results:**

Of 4319 patients, 57 (1.3%) developed PJI. Lower serum albumin was associated with higher infection odds (odds ratio (OR) 0.87 per 0.1 g/dL, 95% confidence interval (CI) 0.78–0.97; *p* = 0.010); blood glucose with increased risk (OR 1.01 per mg/dL, 95% CI 1.00–1.02; *p* = 0.014); and LBT ≤ 35.5 °C with significantly increased risk (OR 2.65, 95% CI 1.40–5.02; *p* = 0.003). The two-factor (area under the curve (AUC) = 0.63) and three-factor (AUC = 0.69) models showed stepwise PJI gradients across cumulative strata; the adjusted multivariable model achieved AUC = 0.71 (95% CI 0.63–0.79).

**Conclusions:**

Serum albumin, blood glucose, and intraoperative LBT are independent modifiable predictors of PJI after primary TKA. A two-factor preoperative model (albumin + glucose) supports preoperative screening; a three-factor model adding LBT provides intraoperative surveillance for structured normothermia protocols. These exploratory cutoffs were derived from a single-center cohort and require external multicenter validation before clinical implementation.

**Graphical Abstract:**

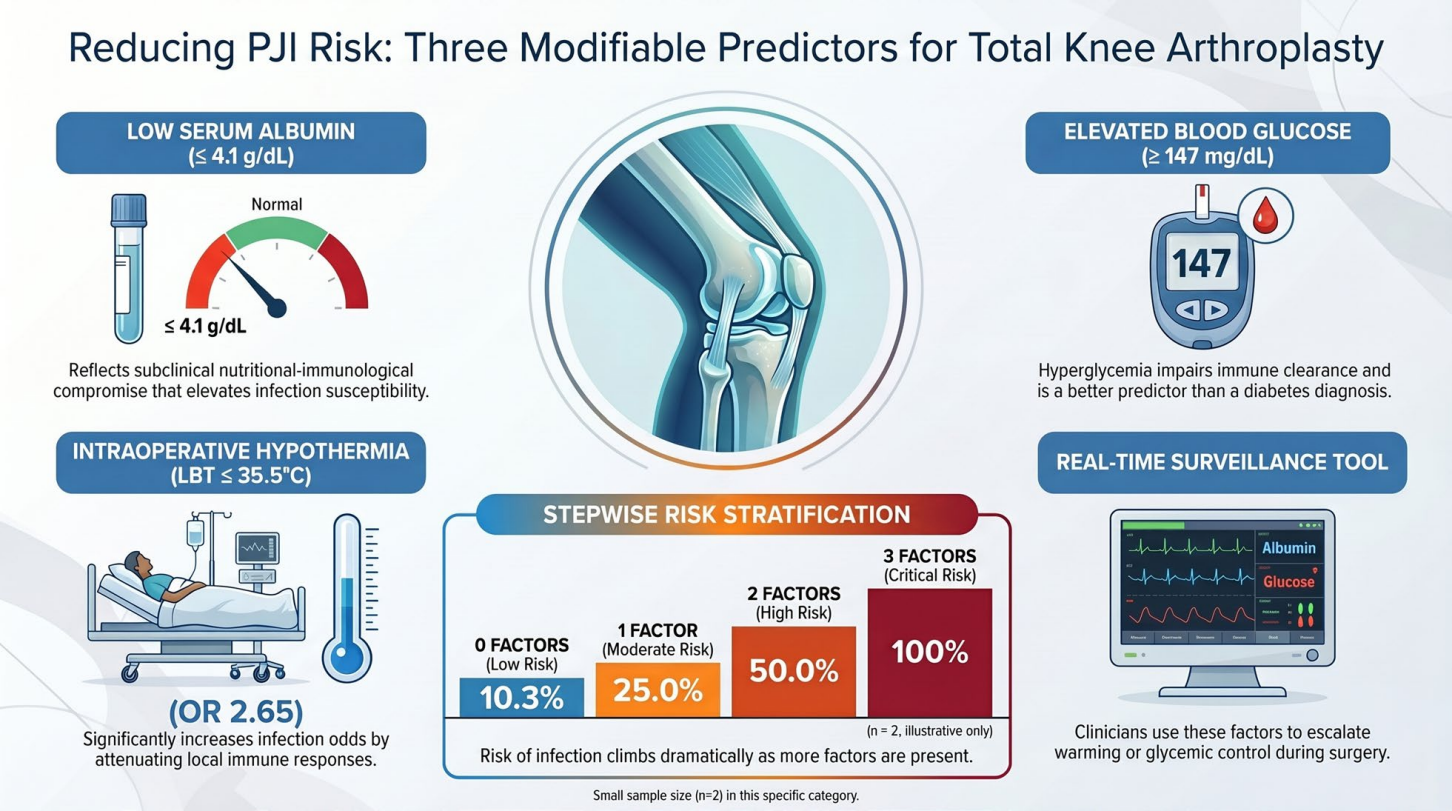

**Supplementary Information:**

The online version contains supplementary material available at 10.1186/s43019-026-00335-3.

## Introduction

Total knee arthroplasty (TKA) is one of the most frequently performed elective orthopedic procedures worldwide [1, 2]. Periprosthetic joint infection (PJI) remains among the most serious and resource-intensive adverse events after primary TKA, with incidence varying across registries depending on patient characteristics and surveillance methodology [3]. Because affected patients commonly require revision surgery, prolonged antimicrobial therapy, and extended hospitalization, infection-related costs are several-fold higher than those of primary procedures [4–6]. These considerations highlight the importance of identifying modifiable perioperative risk factors that can be addressed before implantation.

Numerous host and surgical variables have been associated with PJI risk [7]. However, many established predictors, such as age or baseline comorbidity burden, are not modifiable at the time of surgery. In contrast, several routinely measured perioperative physiological parameters are potentially correctable. Preoperative nutritional status represents one such domain. Serum albumin, a routinely measured marker of protein reserve and systemic inflammatory status, provides an accessible reflection of host physiological resilience [8]. Lower serum albumin has been associated with increased postoperative complications, surgical site infection, and impaired wound healing after total joint arthroplasty [9].

Perioperative glycemic control constitutes a second actionable domain. Hyperglycemia impairs neutrophil function, reduces oxidative killing capacity, and disrupts inflammatory pathways critical for early bacterial clearance [10, 11]. Stress-mediated hyperglycemia is common in surgical populations and may represent an under-recognized contributor to infection susceptibility [12, 13]. Perioperative glucose level, rather than the binary diabetes label, may more accurately reflect infection-relevant biological vulnerability [14, 15].

Intraoperative hypothermia, defined as a core temperature below 36 °C, represents a third modifiable physiological exposure. Hypothermia induces peripheral vasoconstriction, reduces tissue oxygen tension, and attenuates innate immune responses, thereby potentially impairing local bacterial clearance at the implant–tissue interface [16]. Early clinical investigations demonstrated that maintenance of perioperative normothermia reduces surgical-wound infection rates [17], though more recent large non-cardiac surgical trials have reported heterogeneous findings [18]. As an intraoperative parameter under direct anesthetic control, lowest body temperature (LBT) is uniquely suited to function as a real-time surveillance instrument rather than a preoperative predictor.

While several validated PJI risk-stratification tools exist (e.g., the Mayo PJI risk score and International Consensus Meeting (ICM)-derived calculators), most are weighted toward unmodifiable host factors such as age, sex, comorbidity burden, and surgical complexity, limiting their utility for triggering perioperative optimization protocols [19, 20]. The clinical gap therefore lies in a parsimonious, biologically coherent framework built exclusively on routinely measured, perioperatively modifiable physiological parameters that can directly inform timing of surgery, glycemic optimization, and intraoperative warming strategy. While nutritional compromise, hyperglycemia, and hypothermia have each been examined independently, PJI is widely recognized as a multifactorial complication [7]. Accordingly, this single-center retrospective case-matched cohort study evaluated the independent associations of serum albumin, preoperative blood glucose, and intraoperative LBT with PJI within 1 year after primary TKA. We hypothesized that these three modifiable perioperative parameters independently predict PJI and, when combined, provide useful risk stratification for targeted perioperative optimization. We further assessed whether a composite scoring model based on data-driven cutoffs could identify a clinically meaningful, stepwise gradient in infection risk across cumulative strata, presented as both a two-factor preoperative model and a three-factor perioperative model.

## Materials and methods

### Study design and setting

This was a single-center retrospective case-matched cohort study conducted at a tertiary academic medical center. Ethical approval was obtained from the Institutional Review Board of our institution (approval number withheld for blinding). The requirement for informed consent was waived by the IRB given the retrospective nature of the study and use of de-identified clinical data. All procedures were conducted in accordance with the Declaration of Helsinki.

### Study population

**Fig. 1:**
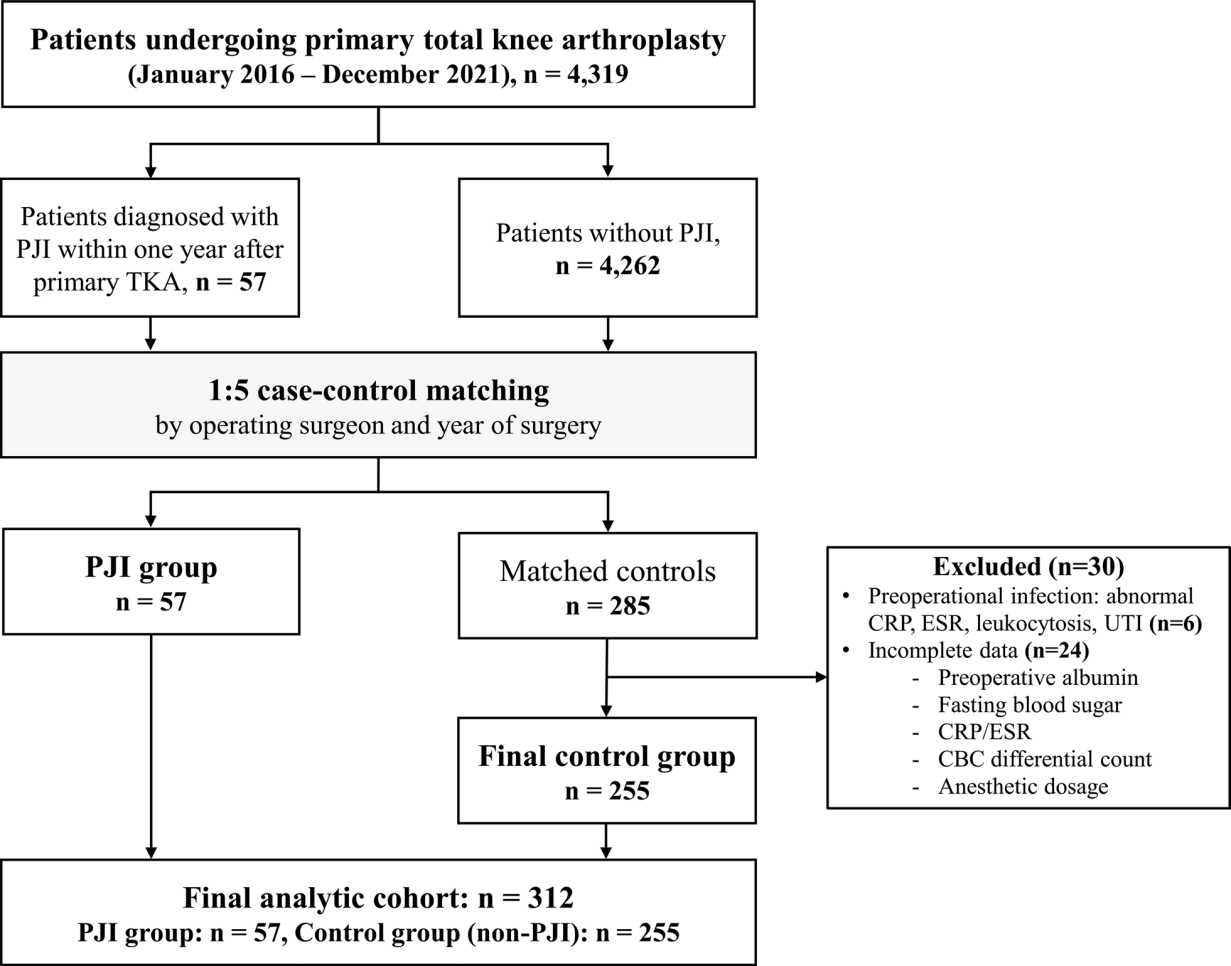
Flowchart of study design and patient selection for a case-matched cohort study of periprosthetic joint infection following primary total knee arthroplasty. A total of 4319 patients who underwent primary total knee arthroplasty (TKA) were initially screened. Collectively, 57 patients who developed periprosthetic joint infection (PJI) within 1 year postoperatively were identified as cases. Controls were selected at an intended 1:5 ratio matched by operating surgeon and year of surgery, yielding 285 candidate controls. After exclusion of 30 controls owing to abnormal preoperative inflammatory markers (elevated C-reactive protein [CRP], erythrocyte sedimentation rate [ESR], leukocytosis, or urinary tract infection; *n* = 6), or incomplete medical records (*n* = 24), a final matched cohort of 312 patients (57 cases and 255 controls) was retained for analysis. Abbreviations: *CRP* C-reactive protein, *ESR* erythrocyte sedimentation rate, *PJI* periprosthetic joint infection, *TKA* total knee arthroplasty, *UTI* urinary tract infection

We retrospectively identified all 4319 adult patients who underwent primary TKA at our institution between 2016 and 2021. PJI within 1 year occurred in 57 of these patients, yielding an overall PJI incidence of 1.3%; the remaining 4262 patients were infection-free and formed the pool from which matched controls were subsequently selected (Fig. 1; Table 1). Patients meeting inclusion criteria (complete preoperative laboratory data, intraoperative temperature monitoring records, and minimum 1-year postoperative follow-up) were screened for the occurrence of PJI within 1 year after the index procedure. In total, 57 patients who developed PJI were designated as cases, and matched controls were drawn from the remaining infection-free cohort at a 1:5 ratio by operating surgeon and year of surgery. After exclusion of 30 candidate controls owing to abnormal preoperative inflammatory markers, active urinary tract infection, or incomplete medical records, the final analytic cohort comprised 312 patients (57 PJI cases, 255 controls; Fig. 1). Frequency matching by surgeon and year was selected to control for intersurgeon practice variation and longitudinal shifts in institutional infection-prevention protocols, isolating patient-level physiological factors as the principal sources of between-group variation. As frequency matching at the group level (rather than individual case–control pairing) does not strictly require conditional logistic regression, standard logistic regression was used for the primary analysis with bootstrap-based sensitivity analyses to verify robustness [21].

**Table 1:** Baseline characteristics of case-matched patients following primary total knee arthroplasty, stratified by periprosthetic joint infection status (*n* = 312)

Variable (unit)	Control group (*n* = 255)	PJI group (*n* = 57)	*P*-value
Demographics			
Age (year)	70.0 (66.0–75.0)	70.0 (66.0–76.0)	0.911
Sex (female)	199 (78.0%)	40 (70.2%)	0.205
Sex (male)	56 (22.0%)	17 (29.8%)	
Body mass index (kg/m^2^)	27.2 (24.6–30.1)	27.2 (25.0–31.3)	0.275
ASA score I & II	131 (51.4%)	26 (45.6%)	0.432
ASA score III	124 (48.6%)	31 (54.4%)	
Comorbidities			
Diabetes mellitus	75 (29.4%)	21 (36.8%)	0.272
Hypertension	192 (75.3%)	46 (80.7%)	0.386
Chronic kidney disease	32 (12.5%)	8 (14.0%)	0.762
Coronary artery disease	16 (6.3%)	1 (1.8%)	0.328
Heart failure	4 (1.6%)	0 (0.0%)	1.000
Chronic obstructive pulmonary disease	4 (1.6%)	3 (5.3%)	0.117
Cerebral stroke	20 (7.8%)	5 (8.8%)	0.789
Autoimmune disease	15 (5.9%)	3 (5.3%)	1.000
Rheumatoid arthritis	10 (3.9%)	3 (5.3%)	0.712
Malignancy	34 (13.3%)	8 (14.0%)	0.888
Peripheral vascular disease	4 (1.6%)	3 (5.3%)	0.117
Smoking (recent use)	10 (3.9%)	4 (7.0%)	0.296
Steroid use	24 (9.4%)	5 (8.8%)	0.880
Medical history of knee			
Prior intraarticular injection	70 (27.5%)	9 (15.8%)	0.067
Prior knee ligament surgery	5 (2.0%)	3 (5.3%)	0.164
Prior septic arthritis	1 (0.4%)	0 (0.0%)	1.000
Preoperative laboratory values			
Serum albumin (g/dL)	4.50 (4.30–4.70)	4.36 (4.16–4.59)	0.001^*^
Blood glucose (mg/dL)	107.0 (95.0–128.0)	110.0 (97.5–144.0)	0.127
Hemoglobin (g/dL)	12.9 (11.9–13.7)	12.7 (11.9–13.7)	0.830
White blood cell count (× 10^3^/μL)	6.90 (5.70–8.10)	6.80 (5.50–7.90)	0.900
C-reactive protein (mg/dL)	1.72 (0.74–3.81)	2.09 (1.04–3.90)	0.325
Erythrocyte sedimentation rate (mm/h)	16.0 (10.0–28.0)	21.0 (10.0–37.0)	0.237
Lymphocyte (%)	28.1 (22.7–32.9)	26.1 (21.7–33.7)	0.583
Intraoperative factors			
LBT (°C)	35.9 (35.6–36.2)	35.7 (35.4–36.1)	0.030*
LBT < 36 °C	132 (51.8%)	38 (66.7%)	0.055
LBT ≤ 35.5 °C	60 (23.5%)	25 (43.9%)	0.002*
Intraoperative hypotension	18 (7.1%)	4 (7.0%)	1.000
General anesthesia	249 (97.6%)	51 (89.5%)	0.010*
Inhaled anesthetic (mL/kg/h)	0.21 (0.17–0.26)	0.18 (0.14–0.28)	0.116
Intravenous fluid (mL/kg/h)	2.85 (2.17–3.44)	2.33 (1.77–3.15)	0.011^*^
Blood transfusion	3 (1.2%)	2 (3.5%)	0.227
Surgery duration (h)	3.0 (2.77–3.42)	3.05 (2.83–3.33)	0.499
Tranexamic acid use	241 (94.5%)	50 (87.7%)	0.079
Morphine dosage (mg)	15.0 (10.0–20.0)	15.0 (10.0–15.5)	0.240
Intraarticular injection			
None	120 (47.1%)	23 (40.4%)	0.326
Morphine	1 (0.4%)	0 (0.0%)	
Transamine	66 (25.9%)	12 (21.1%)	
Morphine + transamine	68 (26.7%)	22 (38.6%)	
Postoperative outcomes			
Postoperative nausea and vomiting	8 (3.1%)	2 (3.5%)	1.000
Length of stay (day)	4.0 (3.0–4.0)	4.0 (4.0–5.0)	< 0.001^*^

Data are presented as median (interquartile range) or number (percentage). ^*^Statistically significant (*p* < 0.05). Standardized mean differences (SMDs) for 12 representative baseline variables are provided in Supplementary Table S1. Abbreviations: *ASA* American Society of Anesthesiologists, *LBT* lowest body temperature, *PJI* periprosthetic joint infection

Patients were excluded if they had: (1) incomplete preoperative laboratory records; (2) a history of prior joint infection at the operative site; (3) concurrent active systemic infection at the time of surgery; (4) revision arthroplasty procedures; or (5) immunocompromising conditions not captured in the standardized dataset, such as HIV-positive status.

### Outcome definition

The primary outcome was the occurrence of PJI within 1 year following the index arthroplasty procedure. PJI was defined according to the 2018 ICM criteria [22, 23], incorporating clinical signs of wound inflammation, joint aspiration culture results, synovial fluid white blood cell counts, and histopathological findings when available. The 1-year window was selected as the predominant convention in contemporary PJI literature and registry-based surveillance studies [22, 23], capturing both early postoperative and acute hematogenous infections temporally linked to the index procedure under the unified 2018 ICM definition. Diagnosis was confirmed by the treating orthopedic surgeon and verified through chart review by two independent investigators.

### Study variables

Demographic variables collected included age, body mass index (BMI), and American Society of Anesthesiologists (ASA) physical status classification. Comorbidities documented at the time of surgery included diabetes mellitus, hypertension, chronic kidney disease, coronary artery disease, heart failure, chronic obstructive pulmonary disease, cerebral stroke, autoimmune disease, rheumatoid arthritis, active smoking status, and chronic corticosteroid use. Relevant medical history included prior intraarticular injection history, prior knee ligament surgery, and history of septic arthritis.

Preoperative laboratory values collected within 7 days before surgery included serum albumin (g/dL), hemoglobin (g/dL), white blood cell count (WBC, × 10^3^/μL), C-reactive protein (mg/dL), and erythrocyte sedimentation rate (ESR, mm/h); fasting blood glucose (mg/dL) was measured the day before surgery. Serum albumin was recorded as the primary nutritional marker:

Serum albumin (g/dL) was analyzed both as a continuous variable and dichotomized at a clinically anchored threshold (≤ 4.1 g/dL)

Intraoperative variables included the lowest recorded body temperature (°C), anesthesia type (general versus regional), use of tranexamic acid, total intraoperative morphine dose (mg), and intraarticular drug injection type.

### Composite risk model construction

Two composite risk models were constructed from prespecified cutoff values: the serum albumin threshold was anchored to established hypoalbuminemia thresholds in arthroplasty, whereas the glucose and LBT thresholds were derived by maximizing the Youden index (J = sensitivity + specificity − 1) in receiver operating characteristic (ROC) analysis. The two-factor preoperative model (model D) incorporated serum albumin ≤ 4.1 g/dL and preoperative blood glucose ≥ 147 mg/dL. The three-factor perioperative model (model E) additionally incorporated intraoperative LBT ≤ 35.5 °C. The serum albumin threshold (≤ 4.1 g/dL) was anchored to prior arthroplasty literature, demonstrating an association between lower preoperative nutritional index and increased postoperative adverse outcomes [24–26]. The blood glucose cutoff of ≥ 147 mg/dL was derived from ROC analysis, approximating commonly referenced perioperative glycemic alert thresholds consistent with evidence linking elevated perioperative glucose to adverse surgical outcomes [12, 27]. This threshold was selected as a pragmatic alert level within the commonly recommended inpatient glycemic target range (140–180 mg/dL), rather than as a treatment initiation threshold. The LBT cutoff of ≤ 35.5 °C was derived from ROC analysis; while below the widely accepted 36 °C threshold for inadvertent perioperative hypothermia, this value captures a more severe thermal exposure associated with attenuated immune defense [16]. These ROC-derived cutoffs were optimized within the present dataset and are therefore presented as exploratory thresholds requiring external validation; they are intended as decision-support triggers rather than definitive diagnostic parameters.

### Statistical analysis

Continuous variables are presented as median with interquartile range [IQR] and compared using the Mann–Whitney *U* test, given non-normal data distributions confirmed by Shapiro–Wilk testing. Categorical variables are expressed as frequency and percentage and compared using Pearson’s chi-squared test or Fisher’s exact test as appropriate. Effect sizes were reported as Cliff’s delta for continuous outcomes and Cohen’s *h* for binary outcomes. Standardized mean differences (SMDs) for 12 representative baseline variables spanning all characteristic domains are presented in Supplementary Table S1 to permit transparent assessment of between-group balance.

Multivariable binary logistic regression was performed to identify independent predictors of PJI, using a parsimonious model with seven prespecified covariates (events-per-variable [EPV] = 8.1, approaching the recommended threshold of ≥ 10): serum albumin, preoperative blood glucose, intraoperative LBT (≤ 35.5 °C), age, BMI, ASA physical status, and C-reactive protein. Covariates were prespecified on the basis of biological plausibility and established PJI literature [14, 15, 27–30]; collinearity was assessed by variance inflation factor (VIF), and model calibration was assessed by the Hosmer–Lemeshow goodness-of-fit (GOF) test. To address potential clustering effects from frequency matching, 95% confidence intervals (CIs) were additionally derived from 2000-iteration nonparametric bootstrap resampling. Restricted cubic splines (RCS) with three and four knots were used to assess potential nonlinearity in the LBT–PJI association; departure from linearity was tested by likelihood ratio. Results are reported as odds ratios (ORs) with 95% CIs.

ROC curve analysis was performed to evaluate the discriminative performance of five models: three individual predictors (models A–C: serum albumin, blood glucose, LBT) and two composite models (model D: albumin + glucose; model E: albumin + glucose + LBT). Area under the curve (AUC) values with 95% CIs were calculated, and pairwise AUC comparisons were performed using the DeLong test. Risk stratification was performed by tabulating observed PJI rates across subgroups defined by the number of concurrent risk factors present (0, 1, and 2 for the two-factor model; 0 to 3 for the three-factor model). Post hoc-achieved power for primary predictors was calculated on the basis of observed effect sizes, sample size (*n* = 312, events = 57), and *α* = 0.05; achieved power was 0.80 for serum albumin (OR 0.87 per 0.1 g/dL), 0.67 for continuous blood glucose (OR 1.01 per mg/dL), and 0.86 for LBT ≤ 35.5 °C (OR 2.54).

All statistical analyses were performed using R software (version 4.3.1; R Foundation for Statistical Computing, Vienna, Austria) and SPSS version 26.0 (IBM Corp., Armonk, NY, USA). A two-tailed *p* value < 0.05 was considered statistically significant for all analyses.

## Results

### Study population and baseline characteristics

Baseline demographic characteristics were comparable between groups; complete distributions of age, sex, BMI, ASA classification, and individual comorbidities are presented in Table 1, with corresponding standardized mean differences (SMDs) in Supplementary Table S1. None of the demographic or comorbidity variables reached statistical significance (*p* > 0.05 for all).

### Preoperative laboratory findings

Preoperative laboratory evaluation demonstrated differences in nutritional markers between groups. Serum albumin was significantly lower in the PJI group (4.36 [4.16–4.59] g/dL) than in the control group (4.50 [4.30–4.70] g/dL; *p* = 0.001). Preoperative blood glucose was numerically higher in the PJI group but did not reach statistical significance in unadjusted comparison (110.0 [97.5–144.0] versus 107.0 [95.0–128.0] mg/dL; *p* = 0.127). Inflammatory and hematological markers (hemoglobin, WBC, CRP, ESR) did not differ significantly between groups (Table 1).

### Intraoperative variables

The lowest recorded intraoperative body temperature was significantly lower in the PJI group (35.7 [35.4–36.1] °C) than in the control group (35.9 [35.6–36.2] °C; *p* = 0.030). The proportion of patients with LBT ≤ 35.5 °C was significantly higher in the PJI group (43.9%) than in the control group (23.5%; *p* = 0.002). General anesthesia was used in 97.6% of patients without infection and 89.5% of those with infection (*p* = 0.01); intravenous fluid volume was lower in the PJI group (2.33 versus 2.85 mL/kg/h; *p* = 0.011). Tranexamic acid use, total intraoperative morphine dose, and intraarticular drug injection type did not differ significantly between groups (Table 1). Postoperative length of stay (LOS) differed between groups despite identical median values: the PJI group showed a heavier right tail (mean 5.04 versus 3.94 days; 95th percentile 7.4 versus 6.0 days; LOS ≥ 5 days: 40.4% versus 17.3%; Mann–Whitney *p* < 0.001). Effect sizes for the three primary predictors were of small-to-moderate magnitude: Cliff’s delta was −0.28 (small) for serum albumin, +0.13 (negligible) for blood glucose, and −0.19 (small) for continuous LBT; Cohen’s *h* was +0.44 (small) for LBT ≤ 35.5 °C and +0.12 (negligible) for ASA III. For the secondary outcome of LOS, Cliff’s delta was +0.35, corresponding to a medium effect.

### Multivariable logistic regression analysis

The parsimonious multivariable model (seven covariates, EPV = 8.1) identified four variables independently associated with PJI (Table 2). Lower serum albumin was independently associated with higher PJI odds (OR 0.87 per 0.1 g/dL decrement, 95% CI 0.78–0.97; *p* = 0.010); preoperative blood glucose with increased odds (OR 1.01 per 1 mg/dL ↑, 95% CI 1.00–1.02; *p* = 0.014); intraoperative LBT ≤ 35.5 °C with markedly increased odds (OR 2.65, 95% CI 1.40–5.02; *p* = 0.003); and BMI with mildly increased odds (OR 1.08 per kg/m^2^ ↑, 95% CI 1.01–1.16; *p* = 0.037). Bootstrap-derived CIs (2000 iterations) closely mirrored analytic intervals, supporting model robustness (Supplementary Table S2). All variance inflation factors were below 1.3 after centering, indicating no meaningful collinearity. The Hosmer–Lemeshow GOF test indicated adequate calibration (*χ*^2^ = 4.67, *df* = 8, *p* = 0.792). The full multivariable model achieved an AUC of 0.71 (95% CI 0.63–0.79).

**Table 2:** Univariate and multivariable logistic regression analyses identifying perioperative risk factors for periprosthetic joint infection after primary total knee arthroplasty (*n* = 312)

Variables (unit)	Median (IQR) or *N* (%)	Univariate analysis		Multivariable analysis	
		OR (95% CI)	*P*-value	OR (95% CI)	*p*-value
Age (year)	70.0 (66.0–75.0)	1.01 (0.97–1.04)	0.754	1.02 (0.98–1.07)	0.400
Body mass index (kg/m^2^)	27.2 (24.7–30.2)	1.05 (0.99–1.12)	0.116	1.08 (1.01–1.16)	0.037^*^
ASA score I & II	157 (50.3%)	1	–	1	–
ASA score III	155 (49.7%)	1.26 (0.71–2.24)	0.432	0.82 (0.43–1.58)	0.557
Serum albumin	4.48 (4.27–4.67)	0.87 (0.80–0.96)	0.005^*^	0.87 (0.78–0.97)	0.010^*^
C-reactive protein (mg/dL)	1.80 (0.80–3.80)	0.99 (0.95–1.03)	0.466	0.97 (0.93–1.02)	0.296
Blood glucose (mg/dL)	107.0 (96.0–130.2)	1.01 (1.00–1.02)	0.017^*^	1.01 (1.00–1.02)	0.014^*^
LBT ≤ 35.5 °C	85 (27.2%)	2.54 (1.40–4.62)	0.002^*^	2.65 (1.40–5.02)	0.003^*^
Model fit (multivariable)	–	Log-likelihood = −134.35; AIC = 284.70; likelihood-ratio *χ*^2^ = 27.98	< 0.001^*^	Hosmer–Lemeshow GOF: *χ*^2^ = 4.67, *df* = 8	0.792
Discriminative performance	–	Adjusted model AUC = 0.71 (95% CI 0.63–0.79, bootstrap)	–	All variance inflation factors < 1.3 (no collinearity)	–

Odds ratios (ORs) with 95% confidence intervals (CIs) are reported for each variable. Multivariable model is the parsimonious seven-covariate logistic regression (events-per-variable = 8.1) with all listed variables entered simultaneously; bootstrap-derived 95% CIs are presented in Supplementary Table S2. ^*^Statistical significance was set at *p* < 0.05. Abbreviations: *AIC* Akaike information criterion, ^ASA^ American Society of Anesthesiologists, *AUC* area under the curve, *CI* confidence interval, *GOF* goodness of fit, *LBT* lowest body temperature, *OR* odds ratio

Sensitivity analysis using continuous LBT in the same adjusted parsimonious model also identified a significant association: each 1 °C decrease in LBT was associated with lower PJI odds (OR 0.52 per 1 °C ↑, 95% CI 0.27–0.99; *p* = 0.048). Restricted cubic splines with three and four knots did not detect significant departure from linearity (LR *p* = 0.51 and 0.07, respectively), indicating that the LBT–PJI association is well captured by either continuous or threshold parameterization. The dichotomized cutoff at 35.5 °C was therefore retained as a clinically interpretable decision rule rather than as a statistically necessary specification.

### ROC analysis and composite model discrimination

**Fig. 2:**
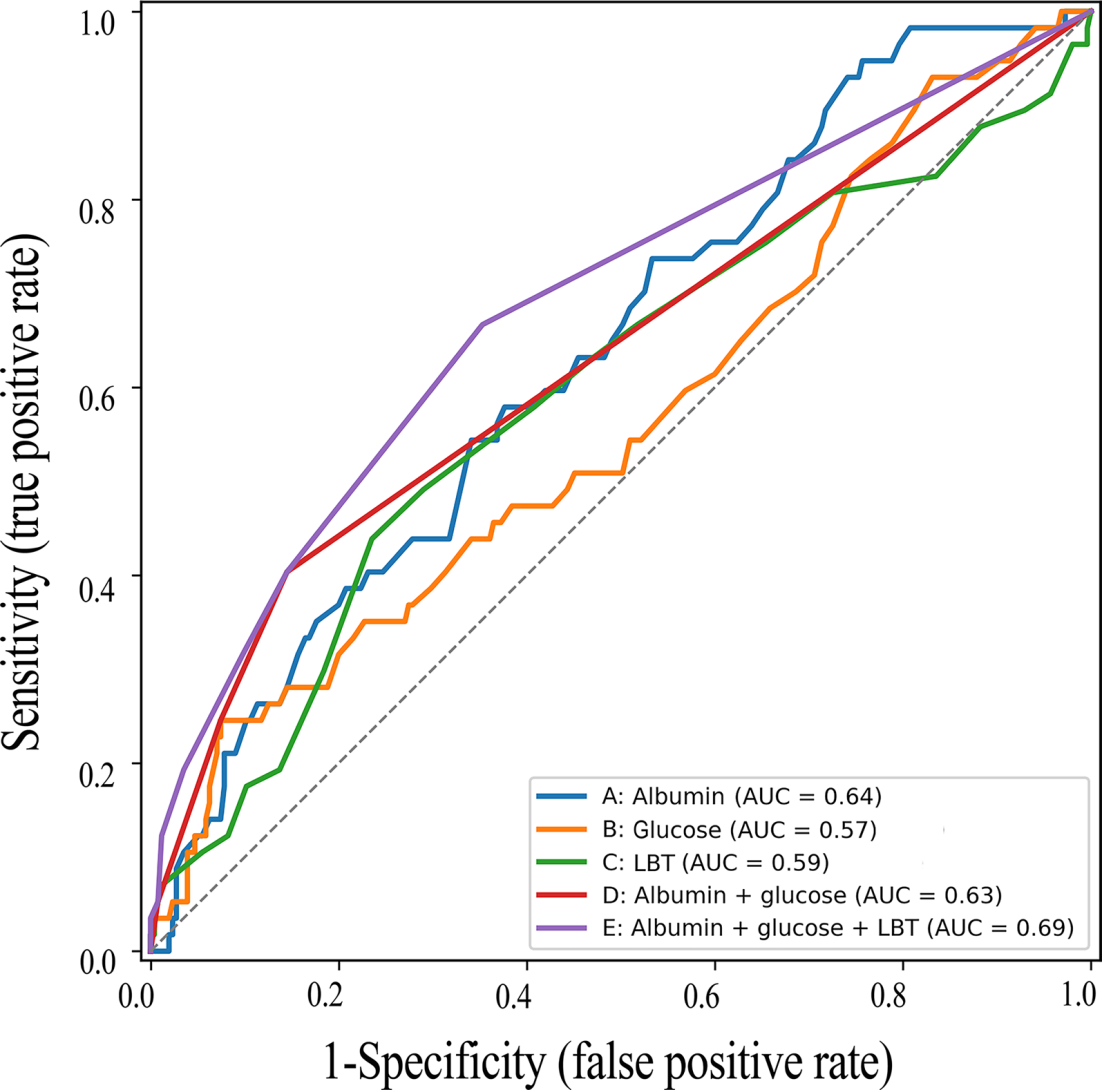
Receiver operating characteristic curves for individual and composite perioperative risk models predicting periprosthetic joint infection after primary total knee arthroplasty. Receiver operating characteristic (ROC) curves are shown for five predictive models: three individual risk factors, serum albumin (model A), preoperative blood glucose (model B), and intraoperative lowest body temperature (model C), and two composite models comprising albumin and blood glucose (model D) and all three variables combined (model E). Optimal cutoff values were determined by maximizing the Youden index. For composite models, the cutoff is expressed as the predicted probability from logistic regression. The diagonal dashed line represents chance-level discrimination (AUC = 0.50). Abbreviations: *AUC* area under the curve, *LBT* lowest body temperature, *PJI* periprosthetic joint infection, *ROC* receiver operating characteristic

ROC analysis of individual predictors demonstrated that serum albumin provided the strongest single-variable discrimination (model A: AUC = 0.64, 95% CI 0.56–0.71, *p* < 0.001), followed by LBT (model C: AUC = 0.59, 95% CI 0.51–0.68, *p* = 0.035), while continuous blood glucose alone did not reach significance (model B: AUC = 0.57, 95% CI 0.48–0.65, *p* = 0.141) (Fig. 2; Table 3). At the prespecified cutoffs (albumin clinically anchored at ≤ 4.1 g/dL; glucose and LBT by the Youden index), serum albumin ≤ 4.1 g/dL was present in 12 patients with PJI (21.1%) versus 20 controls (7.8%); preoperative blood glucose ≥ 147 mg/dL in 14 patients with PJI (24.6%) versus 19 controls (7.5%); and intraoperative LBT ≤ 35.5 °C in 25 patients with PJI (43.9%) versus 60 controls (23.5%) (Table 4). The two-factor preoperative model (model D: albumin ≤ 4.1 g/dL + blood glucose ≥ 147 mg/dL) yielded an AUC of 0.63 (95% CI 0.57–0.71, *p* < 0.001) and the three-factor perioperative model (model E: albumin + glucose + LBT ≤ 35.5 °C) yielded an AUC of 0.69 (95% CI 0.62–0.76, *p* < 0.001). By the DeLong test, the three-factor model significantly outperformed blood glucose alone (model E versus B, *p* = 0.009), whereas the two-factor model did not (model D versus B, *p* = 0.067); model E also significantly outperformed model D (ΔAUC = 0.055, *p* = 0.023) (Table 5).

**Table 3:** Receiver operating characteristic curve analysis of individual and composite perioperative risk models for predicting periprosthetic joint infection after primary total knee arthroplasty

Model	Predictor	AUC	95% CI	*P*-value	Optimal cutoff	Sensitivity	Specificity
A	Albumin	0.64	0.56–0.71	< 0.001^*^	≤ 4.1 g/dL	21.1%	92.2%
B	Glucose	0.57	0.48–0.65	0.141	≥ 147.0 mg/dL	24.6%	92.5%
C	LBT	0.59	0.51–0.68	0.035^*^	≤ 35.5 °C	43.9%	76.5%
D	Albumin + glucose	0.63	0.57–0.71	< 0.001^*^	0.32 (prob)	40.4%	85.5%
E	Albumin + glucose + LBT	0.69	0.62–0.76	< 0.001^*^	0.23 (prob)	66.7%	64.7%
Adjusted	Parsimonious seven-covariate model	0.71	0.63–0.79	< 0.001^*^	–	–	–
Sensitivity	Continuous LBT (adjusted)	0.69	0.62–0.77	0.048^*^	OR 0.52 per 1 °C ↑ (95% CI 0.27–0.99)		

The adjusted parsimonious model row reports discrimination of the full multivariable logistic regression with seven covariates; the sensitivity row reports the continuous-LBT specification for that same adjusted model. ^*^A two-tailed *p* value < 0.05 was considered statistically significant. Abbreviations: *AUC* area under the curve, *CI* confidence interval, *LBT* lowest body temperature

**Table 4:** Distribution of patients meeting for each dichotomous individual perioperative risk factor, stratified by periprosthetic joint infection status

Risk factor	Cutoff (yes/no)	PJI group (*n* = 57)	Control group (*n* = 255)	*P*-value
Serum albumin	≤ 4.1 g/dL	12 (21.1%)	20 (7.8%)	0.006^*^
Blood glucose	≥ 147 mg/dL	14 (24.6%)	19 (7.5%)	< 0.001^*^
LBT	≤ 35.5 °C	25 (43.9%)	60 (23.5%)	0.002^*^

Cutoff values were determined by maximizing the Youden index (J = sensitivity + specificity − 1). Data are presented as the number of patients meeting the cutoff criterion (percentage of group total). ^*^A two-tailed *p* value < 0.05 was considered statistically significant. Abbreviations: *LBT* lowest body temperature, *PJI* periprosthetic joint infection

**Table 5:** Pairwise comparisons of AUC values using the DeLong test among individual and composite models for predicting periprosthetic joint infection after primary total knee arthroplasty

Comparison	AUC 1	AUC 2	Delta AUC	*Z* statistic	*P*-value
Model A (albumin) versus model B (glucose)	0.64	0.57	+0.073	+1.358	0.174
Model A (albumin) versus model C (LBT)	0.64	0.59	+0.044	+0.747	0.455
Model A (albumin) versus model D (albumin + glucose)	0.64	0.63	+0.005	+0.120	0.905
Model A (albumin) versus model E (albumin + glucose + LBT)	0.64	0.69	−0.050	−1.130	0.259
Model B (glucose) versus model C (LBT)	0.57	0.59	−0.029	−0.451	0.652
Model B (glucose) versus model D (albumin + glucose)	0.57	0.63	−0.068	−1.832	0.067
Model B (glucose) versus model E (albumin + glucose + LBT)	0.57	0.69	−0.123	−2.630	0.009^*^
Model C (LBT) versus model D (albumin + glucose)	0.59	0.63	−0.039	−0.663	0.507
Model C (LBT) versus model E (albumin + glucose + LBT)	0.59	0.69	−0.094	−1.928	0.054
Model D (albumin + glucose) versus model E (albumin + glucose + LBT)	0.63	0.69	−0.055	−2.281	0.023^*^

Pairwise AUC comparisons among models A–E were performed using the DeLong test. Each row shows ΔAUC, *Z* statistic, and *p* value

Data are presented as area under the receiver operating characteristic curve (AUC) values and pairwise comparisons using the DeLong test for correlated ROC curves. ΔAUC indicates the difference between two models. ^*^A two-tailed *p* value < 0.05 was considered statistically significant. Abbreviations: *AUC* area under the ROC curve, *LBT* lowest body temperature, *PJI* periprosthetic joint infection, *TKA* total knee arthroplasty, *ΔAUC* difference in AUC

### Risk stratification

**Fig. 3:**
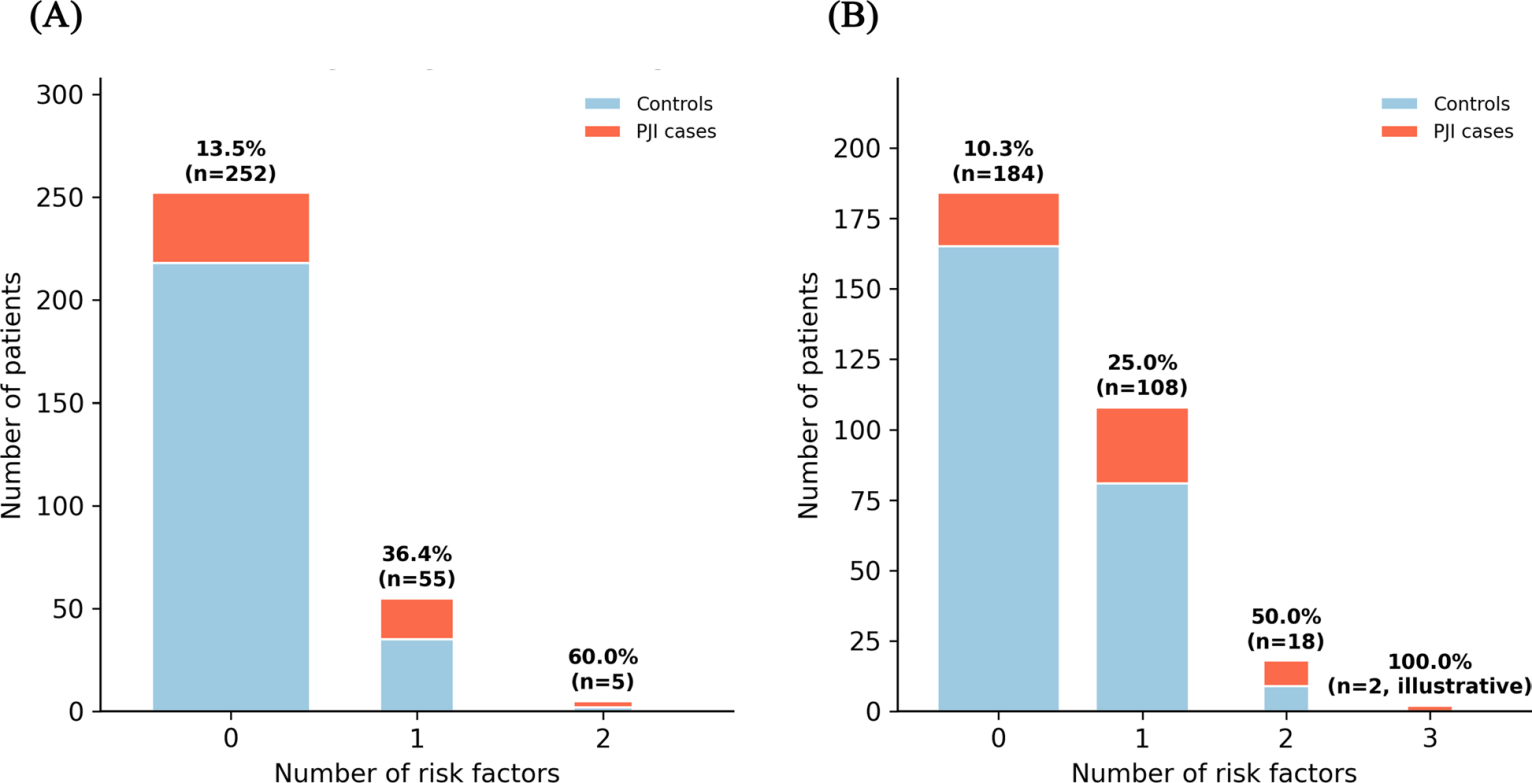
Stepwise risk stratification for periprosthetic joint infection using preoperative and perioperative composite scoring models. **A** Two-factor preoperative model (albumin ≤ 4.1 g/dL + blood glucose ≥ 147 mg/dL; cutoffs determined by Youden index). Stacked bar charts show the distribution of PJI cases and matched controls according to the number of preoperative risk factors present (0, 1, or 2). Observed PJI rates increased progressively across strata: 13.5%, 36.4%, and 60.0% (Spearman *r* = 0.26, *p* < 0.001); *χ*^2^ = 21.74, *p* < 0.001. **B** Three-factor perioperative model (albumin ≤ 4.1 g/dL + blood glucose ≥ 147 mg/dL + LBT ≤ 35.5 °C; cutoffs determined by Youden index). Stacked bar charts show the distribution of PJI cases and matched controls according to the number of perioperative risk factors present (0, 1, 2, or 3). Observed PJI rates increased progressively across strata: 10.3%, 25.0%, 50.0%, and 100% ^†^(Spearman *r* = 0.28, *p* < 0.001); *χ*^2^ = 32.14, *p* < 0.001. *†*The score-3 subgroup comprised only two patients (both with PJI); therefore, the observed 100% PJI rate should be interpreted cautiously and considered illustrative rather than a precise estimate of absolute risk. *CI* confidence interval, *LBT* lowest body temperature, *PJI* periprosthetic joint infection

Risk stratification using model D revealed a stepwise PJI rate gradient across two-factor strata: 13.5% (score 0), 36.4% (score 1), and 60.0% (score 2) (*χ*^2^ = 21.74, *p* < 0.001; Spearman *r* = 0.26, *p* < 0.001). Model E additionally incorporated intraoperative LBT ≤ 35.5 °C, a parameter unavailable before surgery but directly controllable through structured perioperative normothermia protocols, yielding a stepwise pattern across cumulative risk strata: 10.3% (score 0), 25.0% (score 1), 50.0% (score 2), and 100% (score 3) (*χ*^2^ = 32.14, *p* < 0.001; Spearman *r* = 0.28, *p* < 0.001) (Fig. 3). The score-3 stratum contained only two patients; the observed 100% infection rate is statistically unstable and should not be interpreted as a reliable point estimate of absolute risk.

## Discussion

In this single-center case-matched cohort of 312 primary TKA patients with systematic 1-year follow-up using ICM criteria, the overall PJI incidence was 1.3%. Three modifiable perioperative parameters, serum albumin, preoperative blood glucose, and intraoperative LBT, were independently associated with PJI in the parsimonious multivariable model, with adequate calibration (Hosmer–Lemeshow *p* = 0.792) and improved discrimination compared with prior single-variable approaches (adjusted AUC = 0.71). Two composite models were constructed: a two-factor preoperative model (albumin + glucose, AUC = 0.63) and a three-factor perioperative model (albumin + glucose + LBT, AUC = 0.69), each revealing a clear stepwise cumulative risk gradient in observed PJI rates.

### Hypoalbuminemia as a marker of nutritional-immune reserve

Serum albumin is a robust, routinely measured marker of host nutritional and inflammatory status, reflecting protein reserve, immune competence, and overall physiological resilience. Prior arthroplasty literature has consistently linked lower preoperative nutritional and immunological status to adverse postoperative and infection-related outcomes [8, 31]. In our adjusted analysis, lower serum albumin was independently associated with higher PJI odds (OR 0.87 per 0.1 g/dL decrement, 95% CI 0.78–0.97, *p* = 0.010), underscoring the biological relevance of host nutritional-immunological reserve. Our clinically anchored cutoff (serum albumin ≤ 4.1 g/dL) lies above the conventional malnutrition threshold of 3.5 g/dL [31], suggesting that subclinical nutritional-inflammatory compromise elevates infection susceptibility before frank malnutrition is evident, extending prior observations linking preoperative nutritional deficiency to acute postoperative infection risk after total joint arthroplasty [31]. Mechanistically, hypoalbuminemia may reflect reduced oncotic pressure, attenuated immune competence, dysregulated inflammatory resolution, and compromised wound healing at the implant interface [32]. These findings reinforce the value of systematic preoperative serum albumin screening and nutritional optimization as components of perioperative infection prevention pathways [8, 29, 33].

### Perioperative hyperglycemia beyond the diabetes label

Preoperative blood glucose demonstrated an independent association with PJI when analyzed continuously (OR 1.01 per 1 mg/dL increase, *p* = 0.014), whereas a diagnosis of diabetes mellitus alone did not differentiate risk. This observation aligns with evidence suggesting that glycemic state, rather than diagnostic label, more accurately reflects infection-relevant biological vulnerability [15, 28]. Hyperglycemia impairs neutrophil chemotaxis, oxidative burst activity, and microbial clearance, while promoting dysregulated inflammatory responses [32, 34]. These mechanisms are particularly relevant in the context of stress hyperglycemia, where transient perioperative glucose elevations may occur even in patients without established diabetes [12]. Our ROC-derived threshold (≥ 147 mg/dL) aligns with established inpatient glycemic alert thresholds [10]. Together with prior surgical data emphasizing perioperative glycemic control [12, 35], our findings support routine glucose monitoring and targeted optimization regardless of formal diabetes status.

### Intraoperative hypothermia as an independent and modifiable intraoperative risk factor

Intraoperative LBT ≤ 35.5 °C was independently associated with significantly increased odds of PJI (OR 2.65, 95% CI 1.40–5.02, *p* = 0.003) after multivariable adjustment, representing the largest effect estimate among the three identified predictors. Importantly, when LBT was modeled continuously in the same parsimonious framework, each 1 °C decrement was associated with approximately twofold increased PJI odds (OR 0.52 per 1 °C ↑, 95% CI 0.27–0.99, *p* = 0.048), and restricted cubic spline analysis did not reveal significant nonlinearity (LR *p* = 0.51 and 0.07 for three- and four-knot specifications, respectively). Both continuous and threshold parameterizations therefore capture an authentic biological signal; the dichotomized 35.5 °C cutoff is best understood as a clinically interpretable decision rule rather than a statistically required specification. This finding confirms the biological plausibility of intraoperative hypothermia as a modifiable host defense impairment, potentially facilitating bacterial survival at the implant–tissue interface [16]. Observational data from major noncardiac surgery link intraoperative hypothermia with increased infectious complications [36], and early randomized evidence supports the protective effect of perioperative normothermia [17]. Although arthroplasty-specific data have yielded heterogeneous findings [37, 38], the present results suggest that an LBT ≤ 35.5 °C threshold may carry clinically relevant infection risk in TKA. Importantly, unlike serum albumin and blood glucose, which are determined preoperatively, LBT is an intraoperative parameter under direct anesthetic control. This temporal distinction defines the complementary clinical role of our three-factor model: it functions not as a preoperative predictor but as an intraoperative surveillance instrument that enables the perioperative team to identify patients accruing additional thermal risk during surgery and to escalate active warming strategies accordingly. In this framework, orthopedic surgeons and anesthesiologists share responsibility for infection-risk optimization: surgeons may act on preoperative albumin and glycemic screening to defer or modify operative timing, while anesthesiologists implement targeted normothermia protocols intraoperatively on the basis of real-time LBT monitoring [16, 18].

### BMI as a secondary independent predictor

Beyond the three primary predictors, the parsimonious model also identified BMI as an independent risk factor (OR 1.08 per kg/m^2^ ↑, 95% CI 1.01–1.16; *p* = 0.037). This signal, which was masked in the original 15-covariate model owing to over-parameterization (EPV = 3.8), aligns with extensive prior evidence implicating obesity in PJI pathogenesis through impaired wound healing, soft-tissue tension, and altered antibiotic pharmacokinetics [7, 9]. We did not incorporate BMI into the composite scoring framework because it is less amenable to short-term peri-operative modification than serum albumin, glycemia, or thermal control; nevertheless, its persistence as an independent predictor reinforces the multifactorial nature of PJI and suggests that future iterations of structured optimization protocols may benefit from incorporating BMI-based prehabilitation pathways.

Three additional variables demonstrated significant between-group differences but were not retained as independent predictors after multivariable adjustment. The lower rate of general anesthesia in the PJI group (89.5% versus 97.6%; *p* = 0.01) most likely reflects indication bias rather than a causal effect of anesthetic technique, as patients selected for regional anesthesia in observational arthroplasty cohorts often differ in baseline comorbidity and perioperative risk profiles [39]. The association between lower intravenous fluid volume and PJI (2.33 versus 2.85 mL/kg/h; *p* = 0.011) is similarly attributable to confounding by operative complexity and anesthetic practice rather than a direct biological mechanism. Postoperative LOS differed between groups (Mann–Whitney *p* < 0.001) despite identical median values (4 days in both), driven by a heavier right tail in the PJI group (mean 5.04 versus 3.94 days; 40.4% versus 17.3% of patients with LOS ≥ 5 days). Because the median index hospitalization for primary TKA at our institution is 4 days, definitive PJI diagnosis and management are typically completed during readmission rather than the index admission. Accordingly, the observed difference reflects subtle perioperative course variations during the index admission rather than completed infection treatment and should be interpreted as a downstream marker of perioperative complications rather than as evidence of in-hospital PJI management [4, 5].

### Sequential perioperative risk stratification: a two-model framework

A central contribution of this study is the construction of a sequential two-model perioperative risk framework. The two-factor preoperative model (model D: serum albumin ≤ 4.1 g/dL + blood glucose ≥ 147 mg/dL, AUC = 0.63) demonstrated a stepwise PJI gradient of 13.5%, 36.4%, and 60.0% across 0, 1, and 2 risk-factor strata (Spearman *r* = 0.26, *p* < 0.001). The three-factor perioperative model (model E: adding LBT ≤ 35.5 °C, AUC = 0.69) produced a stepwise pattern of 10.3%, 25.0%, 50.0%, and 100% across zero to three strata (Spearman *r* = 0.28, *p* < 0.001), with the score-3 stratum comprising only two patients. The 100% infection rate in this small subgroup is statistically unstable and is presented purely as a directional signal of cumulative risk burden; absolute risk estimates remain unreliable owing to the wide expected CI at this extreme end of the distribution, and the value should not be quoted to individual patients. Model E significantly outperformed model D (ΔAUC = 0.055, *p* = 0.023); because model E’s incremental discrimination derives from intraoperative LBT, model D is retained as the only fully preoperative screening tool, while model E provides complementary intraoperative discrimination. These three variables represent complementary perioperative domains, reduced nutritional-immunological reserve, metabolic stress, and attenuated intraoperative thermal defense, whose convergence may compound infection susceptibility. From a translational standpoint, model D, requiring only two preoperative values available from routine blood work, may assist orthopedic surgeons in identifying high-risk patients for targeted infection prevention strategies, including nutritional optimization and perioperative glycemic control, prior to implantation. Model E serves a complementary intraoperative role: real-time LBT monitoring enables anesthesiologists to identify patients accruing additional thermal risk during surgery and to escalate active warming accordingly (Fig. 3). External validation studies of preoperative PJI prediction models report modest discrimination and variable calibration across cohorts [20], and systematic reviews highlight heterogeneity and limited reproducibility among existing models [19]. In this context, a simple and interpretable multidimensional framework focused on modifiable parameters may offer pragmatic clinical utility beyond what formal discrimination metrics convey.

### Model performance, interpretability, and validation needs

The composite models achieved AUC values of 0.63 (model D, two-factor preoperative) and 0.69 (model E, three-factor perioperative), reflecting modest but meaningful discriminative performance that should not be interpreted as definitive predictive accuracy. The fully adjusted parsimonious multivariable model achieved an AUC of 0.71 (95% CI 0.63–0.79), a discrimination level consistent with established PJI prediction tools developed in much larger registry datasets [19, 20]. On DeLong testing, the three-factor perioperative model significantly outperformed blood glucose alone (model E versus B, *p* = 0.009) and also significantly outperformed the two-factor preoperative model (model E versus D, ΔAUC = 0.055, *p* = 0.023), whereas the two-factor model did not reach significance against glucose alone (model D versus B, *p* = 0.067). Notably, the two-factor composite score (model D, AUC 0.63) did not exceed continuous serum albumin alone (model A, AUC 0.64; ΔAUC = 0.005, *p* = 0.905), reflecting the expected information cost of collapsing a continuous predictor into a dichotomized bedside score rather than any failure of the composite approach. Because this incremental discrimination derives entirely from intraoperative LBT, model D is retained as the only fully preoperative screening instrument—valued for its actionability before incision rather than for maximal discrimination—while model E provides complementary intraoperative surveillance once thermal exposure is known. Model performance in development cohorts characteristically overestimates real-world discriminative ability [40]. Crucially, the practical value of this framework rests not on its absolute discrimination but on its capacity to trigger structured perioperative optimization protocols using inexpensive, routine, and clinically actionable parameters. A composite tool with modest formal discrimination but high interpretability and direct linkage to actionable interventions, nutritional optimization, glycemic correction, and active warming intensification, may provide greater real-world utility than a less interpretable model with higher formal discrimination. Models developed in single-center or narrowly defined populations may show limited transportability owing to case-mix and practice variation [41]. Our findings should therefore be regarded as hypothesis generating, identifying clinically actionable domains that align with established infection-prevention strategies [12, 29] and warrant prospective external validation [42].

### Limitations and future directions

This study has several limitations. First, it is a single-center retrospective analysis: although matching by surgeon and year of surgery effectively controlled for practice variation and temporal shifts in institutional infection-prevention protocols, this design inherently shapes the analytic cohort and may limit external generalizability. In particular, the glucose (≥ 147 mg/dL) and LBT (≤ 35.5 °C) thresholds were optimized within the present matched cohort using the Youden index, whereas the serum albumin threshold (≤ 4.1 g/dL) was clinically anchored; these cutoffs may differ in unmatched, all-comer populations; their performance in such settings requires prospective external validation. The retrospective design carries potential residual confounding from unmeasured factors, including infecting organism characteristics, antibiotic timing, operative complexity, implant variables, and perioperative antimicrobial protocols [32]. The three-factor risk stratum contained only two patients, limiting precision and mandating cautious interpretation. The parsimonious seven-covariate model (EPV = 8.1) was adopted to mitigate overparameterization concerns inherent in 57-event datasets, and bootstrap-based 95% CIs (Supplementary Table S2) confirmed analytic robustness; nonetheless, modest discrimination (AUC 0.63–0.69 for composite models) underscores that the present framework is best regarded as a screening-and-triage instrument rather than a definitive individualized predictor. The absence of patient-level surgeon and year identifiers in the de-identified analytic dataset precluded formal conditional logistic regression; however, given that frequency matching at the surgeon-year stratum level (rather than individual case–control pairing) yields valid inference equivalent to a matched analysis under standard conditions [21], and given the close concordance between analytic and bootstrap intervals, the impact of this limitation on the principal findings is expected to be minimal. Future work should externally validate both composite models in multicenter prospective cohorts, explore whether albumin-targeted nutritional prehabilitation or glycemic optimization reduces PJI incidence, and evaluate whether structured intraoperative warming protocols guided by LBT thresholds improve outcomes in randomized designs [29].

## Conclusions

In this case-matched cohort of patients with primary TKA, three modifiable perioperative parameters, serum albumin, preoperative blood glucose, and intraoperative lowest body temperature, were independently associated with 1-year PJI risk. A two-factor preoperative model (albumin + glucose) identified preoperatively high-risk patients amenable to nutritional and glycemic optimization, while a three-factor perioperative model adding LBT ≤ 35.5 °C extended this framework into the intraoperative period to support real-time anesthesiologist-led active warming strategies. Both models exhibited modest formal discrimination but high clinical interpretability and should be regarded as a pragmatic screening-and-triage framework rather than a definitive individualized predictive algorithm. External multicenter validation remains essential before clinical adoption.

## Supplementary Information

Additional file 1.

## Data Availability

The datasets generated and analyzed during the current study are not publicly available because they contain clinical information that could compromise patient privacy but are available from the corresponding author on reasonable request, subject to approval by the relevant institutional review board.

## Abbreviations

ASA: American Society of Anesthesiologists
AUC: Area under the curve
BMI: Body mass index
CI: Confidence interval
CRP: C-reactive protein
EPV: Events per variable
ESR: Erythrocyte sedimentation rate
GOF: Goodness of fit
ICM: International Consensus Meeting
IQR: Interquartile range
LBT: Lowest body temperature
LOS: Length of stay
MME: Morphine milligram equivalent
OR: Odds ratio
PJI: Periprosthetic joint infection
RCS: Restricted cubic spline
ROC: Receiver operating characteristic
SMD: Standardized mean difference
TKA: Total knee arthroplasty
VIF: Variance inflation factor
WBC: White blood cell count
