# Supplementary material for: Perioperative risk stratification for periprosthetic joint infection after primary total knee arthroplasty: a case-matched cohort study incorporating serum albumin, glycemic status, and intraoperative hypothermia
Source: Knee Surg Relat Res. 2026 Jul 10;38:28. doi: 10.1186/s43019-026-00335-3 (PMC13355362; doi:10.1186/s43019-026-00335-3)
Supplement: Supplementary file 1 — Additional file 1. [file 43019_2026_335_MOESM1_ESM.docx]

Supplementary Table S1. Standardized mean differences (SMDs) for baseline characteristics between PJI and control groups (n = 312).

| Variable (unit) | Control group (n=255) | PJI group  (n=57) | SMD | Balance |
| --- | --- | --- | --- | --- |
| Age (year) | 70.0 (66.0–75.0) | 70.0 (66.0–76.0) | −0.05 | negligible |
| BMI (kg/m²) | 27.2 (24.6–30.1) | 27.2 (25.0–31.3) | −0.22 | moderate |
| ASA III (vs I–II) | 124 (48.6%) | 31 (54.4%) | −0.12 | small |
| Albumin (g/dL) | 4.50 (4.30–4.70) | 4.36 (4.16–4.59) | +0.45 | moderate |
| Glucose (mg/dL) | 107.0 (95.0–128.0) | 110.0 (97.5–144.0) | −0.31 | moderate |
| CRP (mg/dL) | 1.72 (0.74–3.81) | 2.09 (1.04–3.90) | +0.13 | small |
| LBT (°C, continuous) | 35.9 (35.6–36.2) | 35.7 (35.4–36.1) | +0.28 | moderate |
| LBT ≤ 35.5 °C | 60 (23.5%) | 25 (43.9%) | −0.44 | moderate |
| Diabetes mellitus | 75 (29.4%) | 21 (36.8%) | −0.16 | small |
| Smoking | 10 (3.9%) | 4 (7.0%) | −0.14 | small |
| LOS (day) | 4.0 (3.0–4.0) | 4.0 (4.0–5.0) | −0.39 | moderate |
| IV fluid (mL/kg/hr) | 2.85 (2.17–3.44) | 2.33 (1.77–3.15) | +0.32 | moderate |

Standardized mean differences (SMDs) computed using pooled standard deviations for continuous variables and Cohen's convention for binary variables. Interpretation: |SMD| < 0.10 negligible, 0.10–0.20 small, 0.20–0.50 moderate, ≥ 0.50 large. Of 12 baseline variables analyzed, 1 (8%) showed negligible imbalance, 4 (33%) small imbalance, 7 (58%) moderate imbalance, and 0 (0%) large imbalance. Matching was performed by surgeon and year only; observed SMD differences therefore reflect targeted biological signals (albumin, glucose, LBT, BMI) rather than residual matching imbalance.

Supplementary Table S2. Bootstrap-derived 95% confidence intervals for the parsimonious multivariable logistic regression model (2,000 iterations).

| Variable | Analytic OR | Analytic 95% CI | Bootstrap OR | Bootstrap 95% CI |
| --- | --- | --- | --- | --- |
| Serum albumin | 0.87 | 0.78–0.97 | 0.87 | 0.74–0.95 |
| Glucose | 1.01 | 1.00–1.02 | 1.01 | 1.00–1.02 |
| LBT ≤ 35.5 °C | 2.65 | 1.40–5.02 | 2.74 | 1.41–5.39 |
| Age | 1.02 | 0.98–1.07 | 1.02 | 0.97–1.07 |
| BMI | 1.08 | 1.01–1.16 | 1.08 | 0.99–1.16 |
| ASA III | 0.82 | 0.43–1.58 | 0.82 | 0.41–1.60 |
| CRP | 0.97 | 0.93–1.02 | 0.97 | 0.91–1.01 |

Bootstrap analyses used 2,000 nonparametric resampling iterations on the full analytic cohort (n = 312). Close concordance between analytic and bootstrap intervals supports model robustness and indicates that potential clustering effects from frequency matching by surgeon and year do not materially affect inference. OR, odds ratio; CI, confidence interval; LBT, lowest body temperature.
